# Evaluation of six methods for external attachment of electronic tags to fish: assessment of tag retention, growth and fish welfare

**DOI:** 10.1111/jfb.14989

**Published:** 2022-01-23

**Authors:** Brendan J. Runde, Jeffrey A. Buckel, Nathan M. Bacheler, Ryan M. Tharp, Paul J. Rudershausen, Craig A. Harms, Tal Ben‐Horin

**Affiliations:** ^1^ Department of Applied Ecology, Center for Marine Sciences and Technology North Carolina State University Morehead City North Carolina USA; ^2^ Southeast Fisheries Science Center National Marine Fisheries Service Beaufort North Carolina USA; ^3^ Department of Clinical Sciences, College of Veterinary Medicine, Center for Marine Sciences and Technology North Carolina State University Morehead City North Carolina USA

**Keywords:** acoustic tags, black sea bass, holding experiment, reef fish, repeated measures, telemetry, transmitters

## Abstract

External attachment of electronic tags has been increasingly used in fish studies. Many researchers have used *ad hoc* attachment methods and provided little or no validation for the assumption that tagging itself does not bias animal behaviour or survival. The authors compared six previously published methods for externally attaching acoustic transmitters to fish in a tank holding experiment with black sea bass *Centropristis striata* (L.). They tracked tag retention, fish growth and external trauma (as a measure of fish welfare) for 60 days. For each of these metrics, the results showed a wide range of responses among tagging treatments. A simple attachment method using a spaghetti tag passed through the dorsal musculature of the fish and tied to the end cap of the transmitter emerged as the preferred option based on high retention, no impact on growth and relatively low detriment to fish welfare. Future field studies using external electronic tagging should consider tag‐related effects that could compromise results when selecting a method for tag attachment.

## 1 INTRODUCTION

Many fish studies using electronic tags have implanted transmitters gastrically or intracoelomically *via* surgery (Jepsen *et al*., 2015). Nonetheless, some authors have opted for external attachment out of either necessity or preference based on study design and logistics (Burnett *et al*., 2020; Burnett *et al*., 2021). For instance, the antenna of a satellite tag must be free to break the surface of the water in order to transmit. Similarly, satellite archival tags are generally designed to detach after a certain period so they (and the data inside them) can be recovered. Recent technological improvements have increased the options available for telemetry studies, including sensors that record information on light (Block *et al*., 2005; Seitz *et al*., 2019), pH (Halfyard *et al*., 2017; Weinz *et al*., 2020), depth (Halttunen *et al*., 2009; Villegas‐Ríos *et al*., 2020), acceleration (Curtis *et al*., 2015), ambient temperature (Gorsky *et al*., 2012) and body temperature (Domeier *et al*., 2019). Some of these sensors (*e.g*., light) require exposure to the environment and thus must be attached externally to function. It has also been shown that external attachment of acoustic transmitters substantially increases detection range (Dance *et al*., 2016). Moreover, external attachment is often much faster than surgery (Jepsen *et al*., 2015) and can be conducted without anaesthesia thereby saving time and money. Finally, some studies must use external tag attachment because surgery would potentially be impractical or bias the results. For example, studies aiming to track fish after they experience barotrauma cannot use surgical implantation because surgery itself would release abdominal gases and perhaps influence post‐release behaviour or survival (Johnson *et al*., 2015). For these reasons, studies using external attachment of electronic tags have become more common in recent years.

**FIGURE 1:**
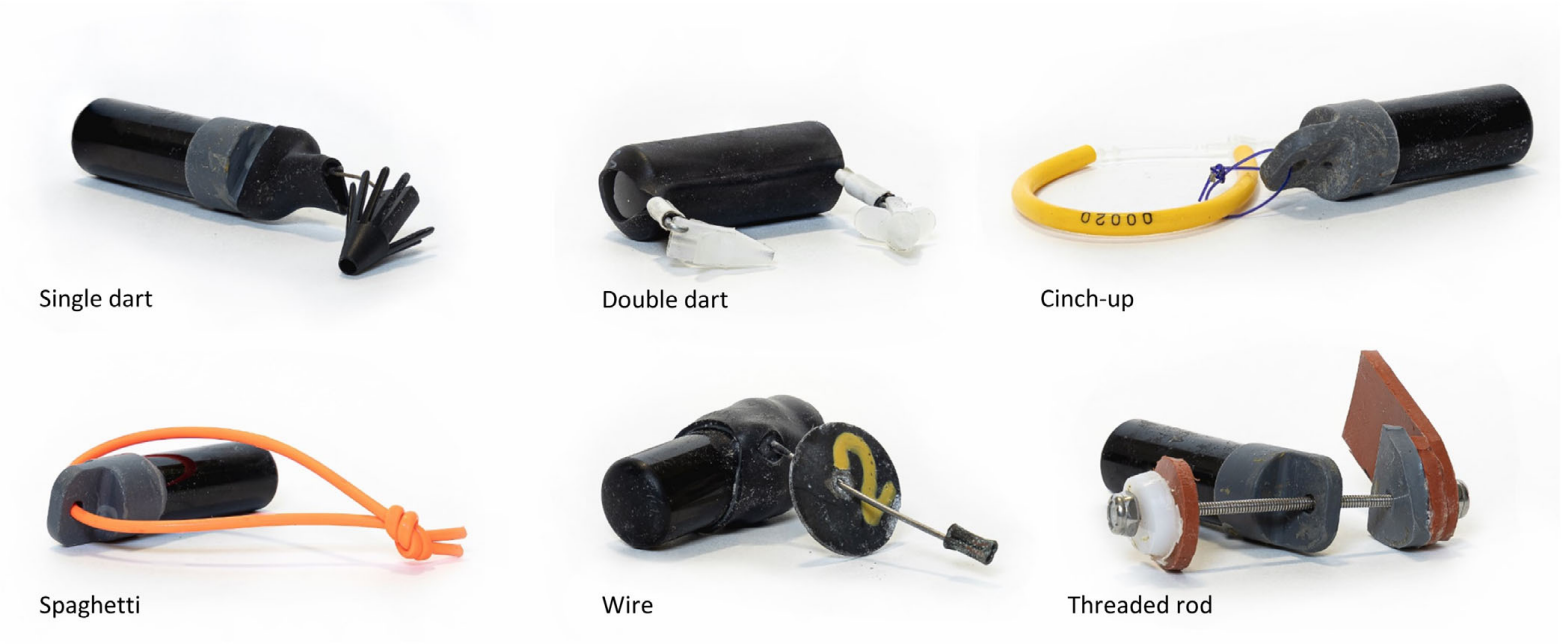
Six methods for attaching acoustic transmitters to fish

Despite the increase in studies using external attachment, there has been no consensus in the fish telemetry literature on how best to attach transmitters. Many studies have developed methodologies with little or no formal evaluation, though some have performed holding studies usually testing tag retention. Telemetry studies typically assume that tagging does not substantially influence behaviour or survival (Jepsen *et al*., 2015; Mellas & Haynes, 1985), so ideal methods for tag affixation should maximize retention over the time‐scale of the study while minimizing adverse physiological effects to the fish. Methods for transmitter attachment have varied widely; several methods are described below and shown in Figure 1.

1. *Single dart*. Domeier *et al*. (2005) used an 8‐prong umbrella‐shaped plastic dart head that can be tethered to a transmitter with metal wire or cable. The dart is typically inserted into the dorsal musculature of the fish, often between the pterygiophores, and has been used in several studies since (*e.g*., Bohaboy *et al*., 2020; Dahl & Patterson, 2020; Dewar *et al*., 2008).
2. *Double dart*. Runde and Buckel (2018), Runde *et al*. (2020) and Wegner *et al*. (2021) affixed transmitters with two small nylon dart tips attached to a length of galvanized steel wire; the transmitter was fixed to the wire with heat shrink and the dart tips were inserted into the dorsal musculature. In these two studies, it was necessary for transmitters to be affixed at both ends because they contained accelerometers and therefore any dangling or swinging of the tag would bias the readings of this sensor.
3. *Cinch‐up*. Eberts *et al*. (2018b) and Eberts *et al*. (2018a) used cinch‐up tags and created a loop passing once through the muscle beneath the dorsal fin. In both studies, the authors incorporated a loop of dissolvable suture material to hold the transmitter to the cinch‐up tag *via* a hole in the transmitter's end cap to promote intentional short‐term loss of the transmitters to minimize the risk of long‐term adverse welfare effects.
4. *Spaghetti*. Capizzano *et al*. (2016) and Capizzano *et al*. (2019) used a method involving the use of a spaghetti‐style tag passed through the dorsal muscle. They threaded the spaghetti tag through the transmitter end cap and tied it with a single overhand knot. Sweezey *et al*. (2020) followed the same spaghetti tag method, but added adhesive to the knot in the spaghetti tag to promote retention.
5. *Wire*. Bacheler *et al*. (2021) wrapped stainless steel wire around the transmitter and used marine‐grade adhesive and heat shrink tubing to hold the wire to the tag. In this method, the exposed end of the wire was sharpened, passed through the dorsal musculature of the fish and held on the opposite side with a large‐diameter aluminium washer and brass crimp. At tagging, the washer and crimp were held firmly against the skin of the fish so the tag was tight against the body.
6. *Threaded rod*. Bohaboy *et al*. (2020) created a method where a 2 mm diameter threaded stainless steel rod was passed through the fish and the transmitter end cap; rubber washers cushioned the fish on either side, and lock nuts held the apparatus together.

These studies represent only some of the variety of attachment methods available to researchers conducting telemetry studies, but to the authors’ knowledge, no direct comparisons of more than two of these methods exist. Indeed, while some of these studies report rates of tag loss (*e.g*., Bohaboy *et al*., 2020), none to the authors’ knowledge empirically examined the impact of tagging on growth and welfare. An experimental comparison of several tag attachment methods would be valuable for designing telemetry studies. Here the authors directly compare the six external attachment methods described above. They provide quantitative results in the form of tag retention and fish growth as well as qualitative and semi‐quantitative results regarding animal welfare. To their knowledge, this study is the first to empirically compare multiple tag attachment methods in this way.

## 2 MATERIALS AND METHODS

### 2.1 Fish capture, tagging and monitoring

The authors conducted the holding study with black sea bass *Centropristis striata* (L.). *C. striata* is an abundant demersal reef fish distributed in the western Atlantic Ocean from Canada to Florida and the Gulf of Mexico, and is important in recreational and commercial fisheries (Musick & Mercer, 1977). Like many species in Serranidae, *C. striata* is a generalist predator and scavenger and associates with hard‐bottom structure. They chose this species given its availability and hardiness in captivity and because of past and planned studies with this species.

*Centropristis striata* were caught *via* hook‐and‐line and fish traps in Onslow and Raleigh Bays, North Carolina, USA, in January and February 2021. Fish were transported in aerated water‐filled coolers to indoor circular 475 l tanks equipped with flow‐through filtered ambient water drawn from and returned to nearby Bogue Sound. Each tank contained habitat enrichment consisting of one concrete block and three 61 cm lengths of 10.2 cm diameter polyvinyl chloride (PVC) pipe. *C. striata* were fed cut Atlantic mackerel *Scomber scombrus* L. and Atlantic herring *Clupea harengus* L. *ad libitum* thrice per week.

After a minimum 1 week acclimation period, *C. striata* were tagged with dummy acoustic transmitters (model V13‐1x; diameter: 13 mm; length: 36 mm; weight in air: 11 g; weight in water: 6.3 g, Innovasea Systems Inc.) on 19 February 2021. The authors chose V13‐1x transmitters because of their popularity in recent fish telemetry studies. Transmitters were attached to each fish using one of six attachment methods (see *Introduction*; Figure 1). Each treatment was intended to match the methods of at least one published article (Table 1). Tags and hardware were soaked in diluted 2% chlorhexidine gluconate solution for disinfection prior to attachment. For tagging, all acclimated *C. striata* were first consolidated into a single tank. Individual fish were selected haphazardly, measured (TL, mm), tagged and weighed [weight in air (g) including tag and hardware]. Time (s) was recorded for each tag application starting when the fish was removed from the holding tank and stopping when the fish was introduced into its destination tank. Treatments were rotated (six treatments plus an untagged control fish), so that the first seven fish went into tank 1, the next seven went into tank 2, *etc*. After six tanks each contained one fish of each treatment plus a control fish (7 fish per tank), the authors tagged an additional 12 fish (2 per treatment) and distributed them into the six tanks in rotation to maintain the same overall stocking density (9 fish per tank). Finally, on 3 March 2021, an additional 6 *C. striata* were tagged (1 per treatment) and introduced into the experimental tanks for a final stocking density of 10 fish per tank. Thus, each tank contained two fish for each of three treatments, one fish for each of the remaining three treatments and one control fish. The stocking density of 10 fish per tank was maintained for the duration of the study. All tagging and husbandry was performed under the approval of North Carolina State University IACUC #19‐608. The six methods tested in this study were chosen because they were either previously used by an author of the present document or were under the consideration of authors of this study for application in upcoming field studies.

**TABLE 1:** Tag attachment treatment names, descriptions and examples of publications employing each method

Treatment	Description	Reference(s)
Single dart	Eight‐prong plastic umbrella dart attached to transmitter end cap *via* nylon‐coated braided wire; wire crimped and covered with heat shrink. Dart inserted into muscle *c*. 2 cm below anterior insertion of dorsal fin.	(Dahl & Patterson, 2020; Dewar *et al*., 2008; Domeier *et al*., 2005)
Double dart	Two nylon darts (Floy FIM‐96) connected with galvanized steel wire; wire crimped at either end, crimps covered with heat shrink. Transmitter (no end cap) attached to wire *via* heat shrink. Darts inserted simultaneously into muscle *c*. 2 cm below anterior insertion of dorsal fin.	(Wegner *et al*., 2021; Runde *et al*., 2020; Runde & Buckel, 2018)
Cinch‐up	Cinch‐up tag (Floy FT‐4) passed through the fish *c*. 2 cm below anterior insertion of dorsal fin *via* hollow stainless steel applicator. Tag cinched through loop of dissolvable polydioxanone suture (PDS; size 0 or 1) tied to transmitter end cap.	(Eberts *et al*., 2018a; Eberts *et al*., 2018b)
Spaghetti loop	Spaghetti tag (Floy FT‐4) passed through the fish *c*. 2 cm below anterior insertion of dorsal fin *via* solid stainless steel applicator. Tag passed through transmitter end cap and tied in single overhand knot. No glue applied.	(Capizzano *et al*., 2016; Capizzano *et al*., 2019; Sweezey *et al*., 2020)
Wire	Stainless steel wire (0.89 mm diameter) wrapped around centre of transmitter (no end cap). Wire affixed to transmitter with marine‐grade adhesive (3 M 5200) and heat shrink. End of wire sharpened and passed through fish *c*. 2 cm below anterior insertion of dorsal fin. Aluminium washer threaded over end of wire and retained with crimp. Extra wire cut.	(Bacheler *et al*., 2021)
Threaded rod	Threaded stainless steel bar (2 mm diameter) sharpened and passed through fish *c*. 2 cm below anterior insertion of dorsal fin. One end of steel bar passed through silicon cushion (to reduce abrasion) and transmitter end cap; the other end passed through polyethylene and silicon discs. Both ends secured with 6.35 mm nylon‐lined stainless steel lock‐nut.	(Bohaboy *et al*., 2020)

*Note*: See Figure 1 for photos of tag attachment methods.

The authors monitored *C. striata* daily for tag loss and fish mortality. When tag loss was discovered, they removed the affected fish and replaced them on the same day with a freshly tagged fish of the same treatment to increase sample sizes and maintain stocking density. They tagged a maximum of two replacement fish per treatment, after which fish that had lost their tag were left in the tank and remained untagged. Every 10 days, they weighed and measured each tagged individual plus the never‐tagged control fish to monitor growth; previously tagged fish were not weighed and measured. During these sessions, they noted any tag‐related lesions or pathologies including scale loss, degree of abrasion and bleeding. External trauma was categorized into four levels: none, mild (abrasion but no scale loss), moderate (scale loss of 1 cm diameter or less; inflamed tissue) or severe (scale loss of more than 1 cm diameter and/or exposed muscle tissue). Starting on day 20 after the first tagging event, they observed signs of infection in some individuals (*i.e*., abscess at the tag insertion site). They swabbed tag‐associated skin lesions from four affected individuals using a commercial bacterial culture collection system (BBL™ CultureSwab™ Plus Collection & Transport System, Copan Italia, Becton, Dickinson and Company Sparks, MD 21152) and submitted samples to a commercial veterinary diagnostic microbiology laboratory (NC State College of Veterinary Medicine, Microbiology and Molecular Diagnostics Laboratory, 1060 William Moore Drive, Raleigh, NC 27607). Swabs were plated for bacterial culture and identification under the U.S. Food & Drug Administration Veterinary Laboratory Investigation and Response Network (https://www.fda.gov/animal-veterinary/science-research/veterinary-laboratory-investigation-and-response-network) “other‐aquatic pathogens” protocol.

The authors concluded the study on 20 April 2021, 60 days after tagging the majority of fish. Tags were removed from most of the still‐tagged fish; these individuals were allowed to heal for a 1 week period prior to release in Bogue Sound. A sub‐set of still‐tagged fish (*n* = 3 per treatment; total *n* = 18) were euthanized with an overdose of tricaine methanesulfonate followed by penetrating captive bolt and pithing. Blood was collected immediately postmortem from the caudal haemal arch with heparinized needle and syringe, loaded in haematocrit capillary tubes, and centrifuged in a haematocrit centrifuge to measure packed cell volume (PCV, *i.e*., erythrocyte volume) and buffy coat (BC) volume (*i.e*., leukocyte volume) as a rough surrogate for white blood cell count (Kerr, 2010). Plasma total solids (TS) was measured by refractometry (RHC‐300ATC clinical refractometer, Ade Advanced Optics, Oregon City, OR 97045) from the capillary tube supernatant. Wedge cross sections of skin and muscle down to the dorsal spinous processes medially and ribs ventrally, no more than 1 cm thick longitudinally, were collected from tag insertion sites and unaffected sites from the same fish (internal control), and fixed individually in 10% neutral buffered formalin for histopathology. Tissue cross sections were trimmed into histology cassettes, embedded in paraffin, decalcified, sectioned at 5 μm and stained with haematoxylin and eosin (NC State College of Veterinary Medicine, Histology Laboratory) for examination by light microscopy. The tissue sections were randomized and evaluated by two observers (T.B‐H. and C.A.H) blinded to treatment. The authors used a six‐point grading scale, developed by Hurty *et al*. (2002), to semi‐quantitatively describe tissue damage associated with the six tagging methods as follows: Grade 0, no visible changes relative to the skin, underlying subcutis and muscle of internal controls; Grade 1, very mild changes, with occasional inflammatory cells and edema present, and the tagging site having minimal fibrosis and well‐organized granulation tissue; Grade 2, mild changes compared to Grade 1; Grade 3, moderate changes, with widespread inflammatory infiltrates and moderate edema in the dermis and subcutis and scattered necrosis of individual muscle bundles; Grade 4, moderately severe changes; and Grade 5, severe changes marked by widespread inflammation, edema and large areas of coagulative necrosis of muscle bundles.

### 2.2 Data analysis

The authors assessed how tag retention varied among treatments with Kaplan–Meier survivorship models. Because of the staggered entry of fish into the study, most individuals were observed for 60 days but some more recently tagged fish were observed for as little as 20 days. For the Kaplan–Meier analysis, fish with retained tags were right‐censored on the day equal to the duration they were in the study. Retention estimates were compared among treatments and evaluated for whether their 95% c.i. overlapped. Kaplan–Meier analyses were performed and visualized in R (R Core Team, 2021) using the packages “survival” (Therneau, 2015) and “survminer” (Kassambara & Kosinski, 2018).

The authors fitted Bayesian mixed regression models to weight data to examine the effect of tag treatment on growth; these models are analogous to repeated measures analysis of variance. They elected to use these models given their flexibility, which was necessary due to the complex study design. Although more traditional models may have resulted in similar findings, they preferred the ability to set priors and handle staggered entries and mixed effects in a Bayesian framework. Tag weights were subtracted from total weights obtained during each 10 day measuring period. For this procedure, the response variable was fish wet weight (g) and candidate models included a range of possible predictor variables in addition to treatment (*Trt*); possible variables were *Day* (fixed effect), Day*Trt two‐way interaction, *Tank* (random effect) and individual (*ID*; random effect).

Models were specified as:

$$\text{Weight}∼\text{Normal}\left(\mu_{i},\sigma \right)$$

$$\mu_{i}=\beta_{0}+\beta_{1}{\mathrm{Day}}_{i}+\beta_{2}{\mathrm{Trt}}_{i}+\beta_{3}{\mathrm{Day}}_{i}*{\mathrm{Trt}}_{i}+\alpha_{{\text{Tank}}_{i}}+\gamma_{{\mathrm{ID}}_{i}}$$

with priors:

$$\beta_{0}∼\text{Normal}\left(300,100\right)$$

$$\beta_{1}∼\text{Normal}\left(0,10\right)$$

$$\beta_{2}∼\text{Normal}\left(0,100\right)$$

$$\beta_{3}∼\text{Normal}\left(0,3\right)$$

$$\alpha_{j}∼\text{Normal}\left(0,\sigma_{\text{tank}}\right)\;\text{where}\;j=1\ldots 6$$

$$\gamma_{j}∼\text{Normal}\left(0,\sigma_{\mathrm{ID}}\right)\;\text{where}\;j=1\ldots 66$$

$$\sigma ∼\text{Exponential}\left(0.1\right)$$

$$\sigma_{\text{Tank}}∼\text{HalfCauchy}\left(10\right)$$

$$\sigma_{\mathrm{ID}}∼\text{HalfCauchy}\left(10\right)$$

Where β terms are variable‐specific coefficients, σ is the overall standard deviation in weights, α_*j*_ is the intercept for tank *j*, γ_*j*_ is the intercept for individual *j*, σ_*Tank*_ is the tank‐specific standard deviation and σ_*ID*_ is the individual‐specific standard deviation. The authors chose lightly informative priors for some terms to apply biologically reasonable constraints and based on prior predictive simulations. They compared fits among models by using leave‐one‐out cross validation information criterion (LOOIC; Vehtari *et al*., 2017). For the best model (lowest LOOIC), importance of partial regression coefficients was evaluated by examining 95% credible intervals and checking whether they contained zero. Models were fit using the R package “brms” (Bürkner, 2017).

For examining fish welfare, they coded the four levels of external trauma (none, mild, moderate, severe) as integers 0–3, respectively. They examined mean and standard error in level of trauma for each tag treatment at each weigh‐in period throughout the study. The PCV, BC, TS and histopathology grades were compared among treatment groups.

### 2.3 Ethical statement

The care and use of experimental animals complied with United States of America animal welfare laws, guidelines and policies as approved by North Carolina State University IACUC #19‐608.

## 3 RESULTS

**FIGURE 2:**
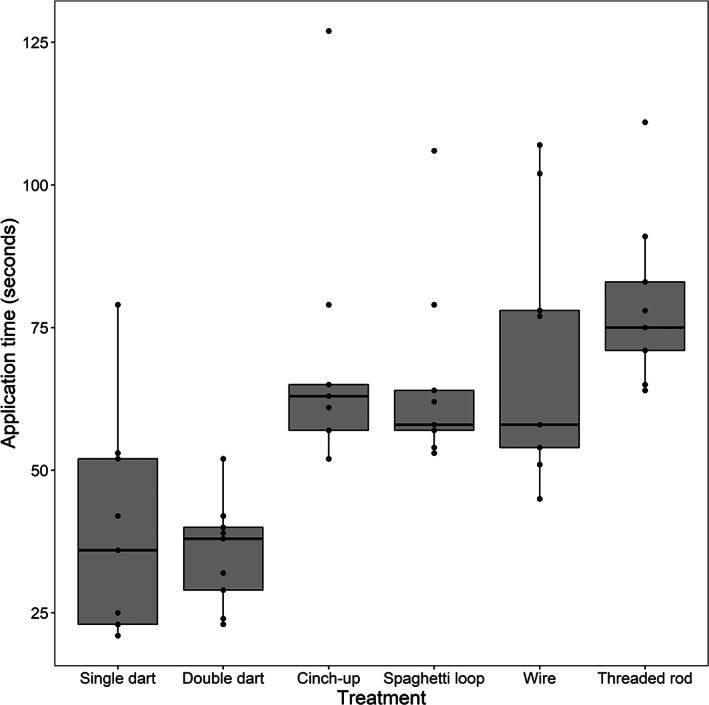
Time (seconds) to apply transmitters to fish in this study using six different attachment methods in *Centropristis striata*. See Table 1 for a description of each method

The first two treatments, single dart and double dart, were applied in the lowest amount of time on average, taking 39 and 35 s respectively (Figure 2). The cinch‐up, spaghetti loop and wire treatments each took *c*. 70 s to apply, on average. The threaded rod took the longest to apply with an average of 79 s. Dry weights of the tag plus hardware for these six treatments were 13.75, 12.93, 13.25, 13.50, 13.75, and 18.40 g, respectively.

**FIGURE 3:**
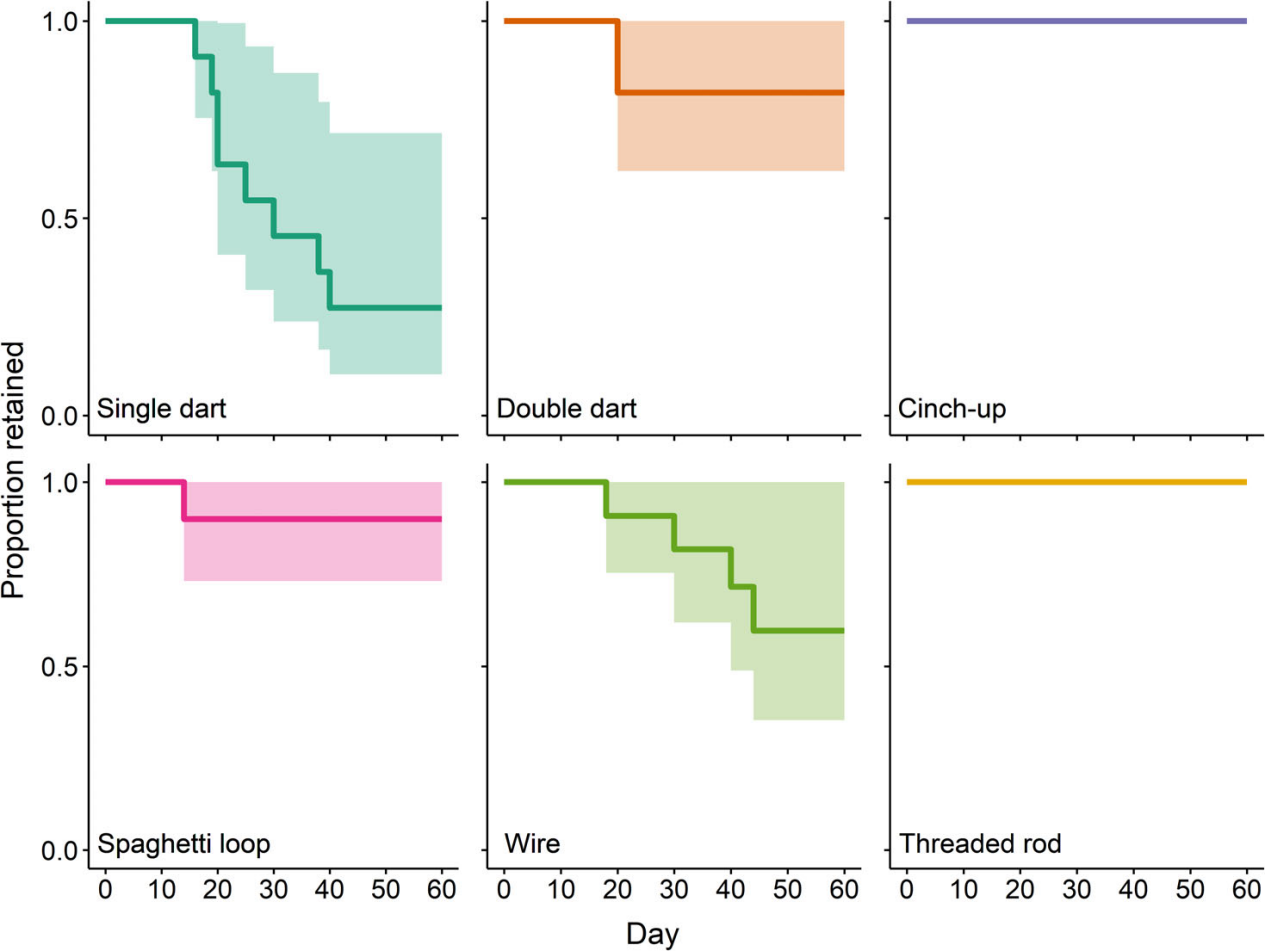
Kaplan–Meier curves for tag retention among six tag attachment methodologies (treatments) in *Centropristis striata*. Shaded regions represent 95% c.i. See Table 1 for a description of each method

None of the *C. striata* in this study died, but multiple fish shed their tags. The authors found the lowest tag retention for the single dart. Of 11 fish tagged with the single dart, 8 shed their tags; tags were lost on days 16, 19, 20, 20, 25, 30, 38 and 40 after tagging. Of 11 fish tagged with the double dart, 2 lost their tags, both on day 20. None of the nine fish tagged with the cinch‐up tag lost their tags. One individual tagged with the spaghetti loop lost its tag on day 14 when the knot in the spaghetti tag came undone; instead of tagging a fresh fish, the authors retied the spaghetti tag to the transmitter of this individual and continued to monitor its growth and welfare. Of 11 fish tagged with the wire treatment, 4 lost their tags (days 18, 30, 40 and 44). None of the nine fish tagged with the threaded rod lost their tags. Kaplan–Meier analyses estimated that the 60 days tag retention for only the single dart differed statistically from 1.00 with a mean estimate of 0.27 (95% c.i. 0.10, 0.72; Table 2; Figure 3). Mean retention estimates for the double dart, spaghetti loop and wire were 0.82, 0.90, and 0.60, respectively; nonetheless, the upper c.i. for all of these methods was 1.00. As no tags attached with the cinch‐up nor threaded rod were lost, mean retention for these methods was estimated to be 1.00.

**TABLE 2:** Kaplan–Meier estimates for 60 day tag mean rates of retention (with 2.5% and 97.5% c.i.) for six external tag attachment methods (“Treatment”)

Treatment	Mean	2.5%	97.5%
Single dart	0.27	0.10	0.72
Double dart	0.82	0.62	1.00
Cinch‐up	1.00	1.00	1.00
Spaghetti loop	0.90	0.73	1.00
Wire	0.60	0.35	1.00
Threaded rod	1.00	1.00	1.00

**FIGURE 4:**
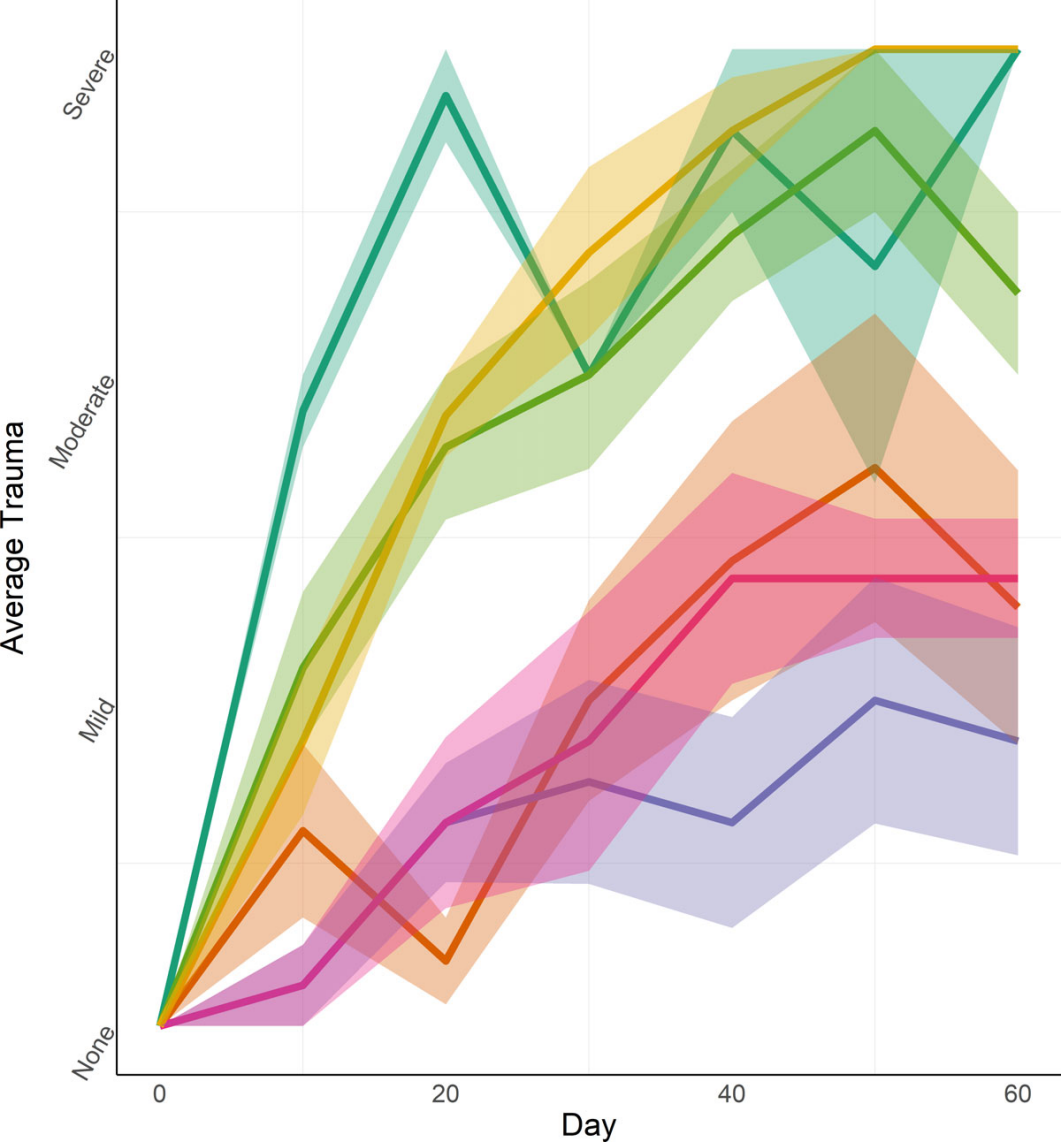
Mean and standard error (shaded regions) of external trauma among tagging treatments in *Centropristis striata*. Trauma was recorded every 10 days throughout the study. The four *y*‐axis categories were coded as 0 (absent), 1 (mild), 2 (moderate) or 3 (severe) for this analysis. See Table 1 for a description of each method. Treatment: (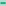) Single dart, (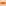) Double dart, (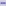) Cinch‐up, (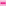) Spaghetti loop, (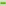) Wire, (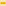) Threaded rod

**FIGURE 5:**
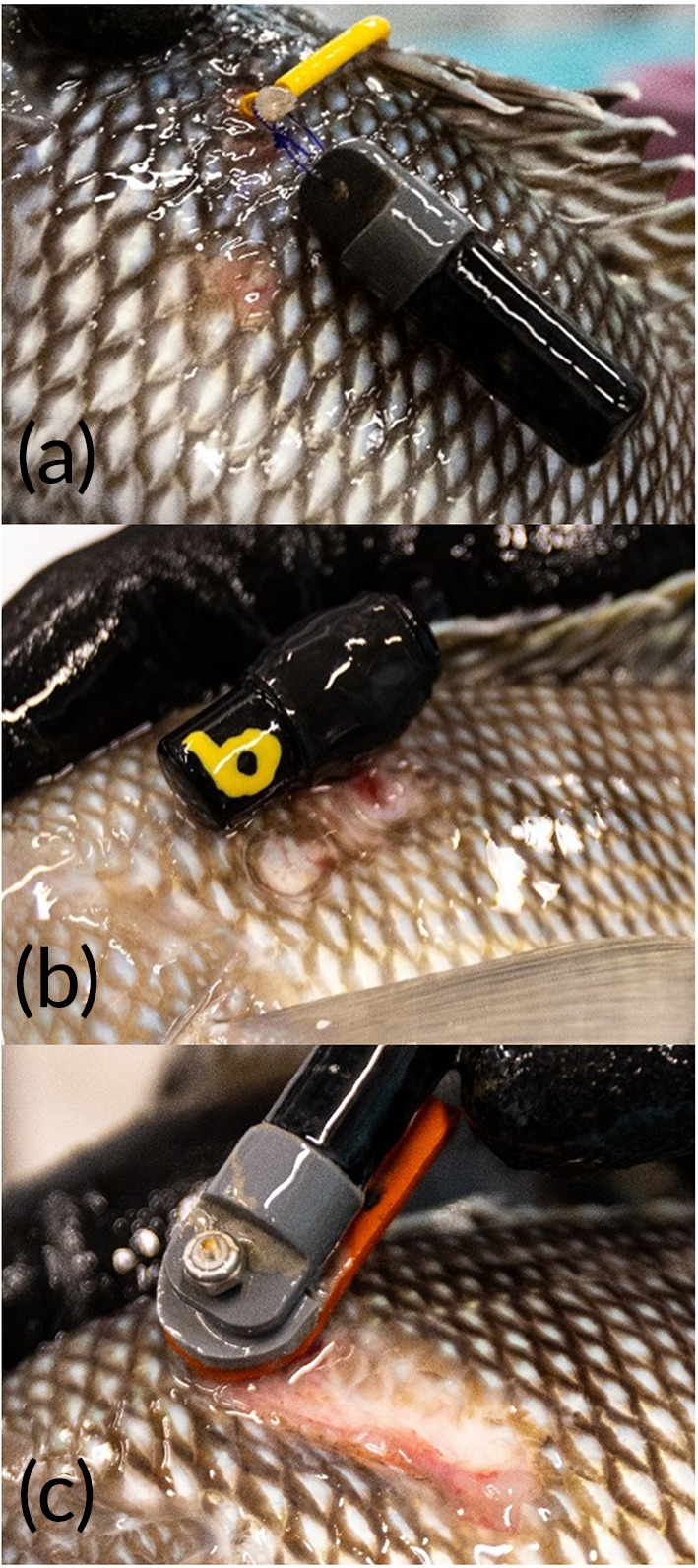
Three degrees of external trauma in tagged *Centropristis striata*: (a) mild, (b) moderate, (c) severe

**FIGURE 6:**
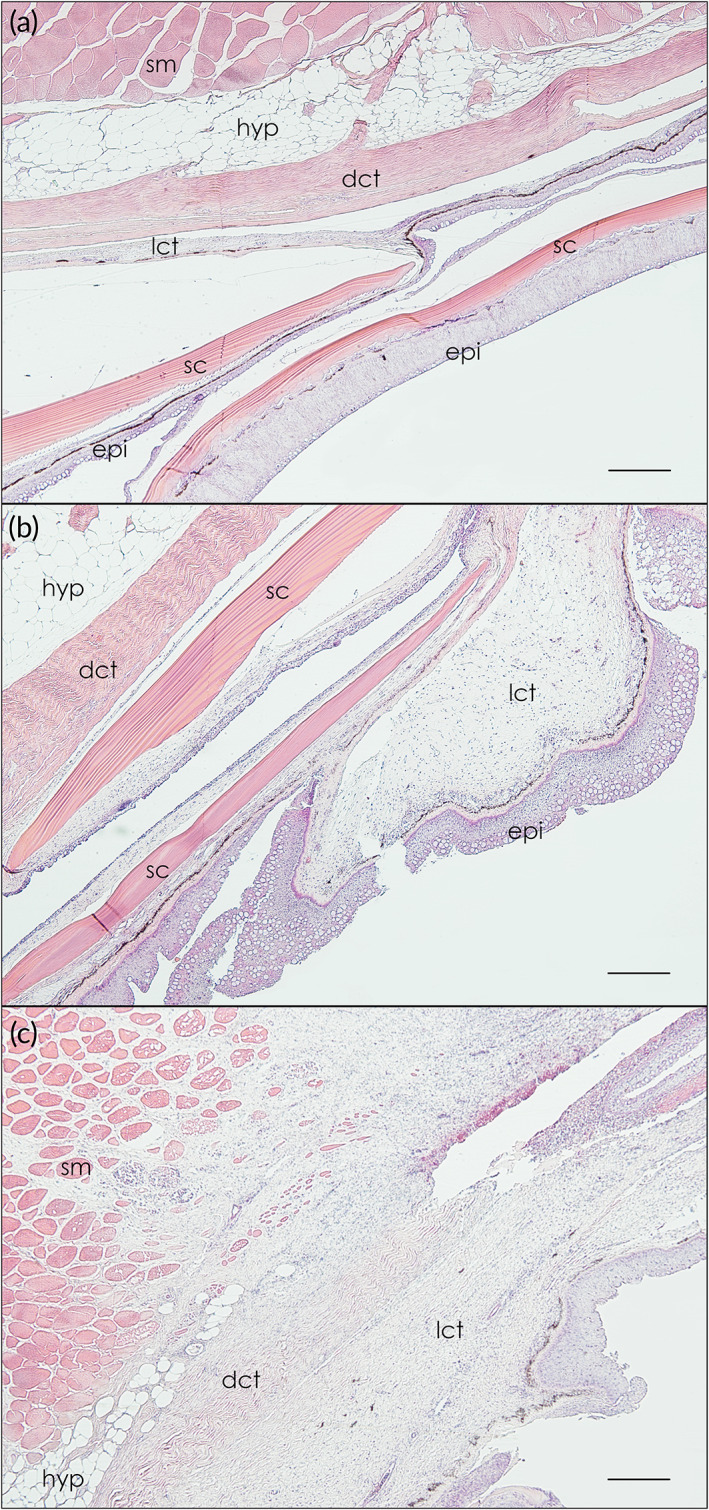
Tissue reactions to tagging. (a) *Centropristis striata* (L.) scales (sc) with intact epidermis (epi) and underlying loose connective tissue (lct), dense connective tissue (dct), hypodermis (hyp) and skeletal muscle bundles (sm). The tissue control shown in (a) represents no tissue damage (Grade 0). (b) Mild to moderate tissue damage (Grade 3) at a tag insertion site highlighting numerous mucus cells in the epidermis and inflammatory cells throughout and enlarging subcutaneous connective tissue. (c) Severe tissue damage (Grade 5) with widespread and focally intense infiltration of mixed inflammatory cells throughout connective tissues and underlying skeletal muscle with necrosis and loss of musculature. The black horizontal scale bar in each panel is 250 μm

All six tagging methods resulted in some level of trauma (Figures 4, 5, 6). The single dart, wire and threaded rod treatments caused moderate to severe trauma on average after approximately 30 days (Figure 5b,c). At day 60, all remaining fish tagged with the single dart and threaded rod had severe trauma. External trauma was the worst for the threaded rod, rising steadily on average to day 50 at which point all nine individuals were classified as severe. The silicon pad that was intended to cushion the tag against the exterior of the fish caused substantial chafing and resulted in scale loss and exposed muscle (Figure 5c). In several individuals tagged with the threaded rod, the washers on the opposite side wore through the scales and into the body of the fish. The double dart, cinch‐up and spaghetti loop each caused much lower levels of external trauma, with the cinch‐up causing only mild trauma on average even at 60 days (Figure 5a). Trauma in fish tagged with the cinch‐up and spaghetti loop was usually characterized by minor chafing from the rim of the tag end cap rubbing on the lateral aspect of the fish. Histologically, samples from areas other than the tagging site showed no tissue damage (Figure 6a). However, all six tagging methods led to clear damage to the epidermis (Figure 6b) and underlying subcutis (Figure 6c) and skeletal muscle (Figure 6c), with all tagging methods having at least one individual scoring Grade 5 (most severe) on the authors’ semi‐quantitative scale.

The rate of obvious abscessation at the tagging site varied by treatment. The authors observed abscesses in 6 of 11 fish for both the single dart and double dart. No abscesses were noted for any fish tagged with the cinch‐up and spaghetti loop. One of the 11 fish tagged with the wire treatment and 1 of the 9 fish tagged with the threaded rod had gross indications of infections. All fish with signs consistent with infection that lost their tag healed (*i.e*., were no longer abscessed) within approximately 1 week. Bacterial cultures of tag attachment wounds of four fish yielded gram‐positive bacteria identified as *Carnobacterium maltaromaticum* and *Lactococcus raffinolactis*, both of which were regarded as environmental opportunists rather than primary pathogens.

No qualitative differences among groups were evident for PCV (combined median 24, range 18%–27%) and plasma TS (combined median 5.6, range 4.4–7.2 g/dl), but BC volumes appeared least for the cinch‐up and spaghetti loop (medians 0, range 0%–1%) and greatest for the single dart (median 4, range 0%–4%) and threaded rod (median 3, range 0%–4%).

**FIGURE 7:**
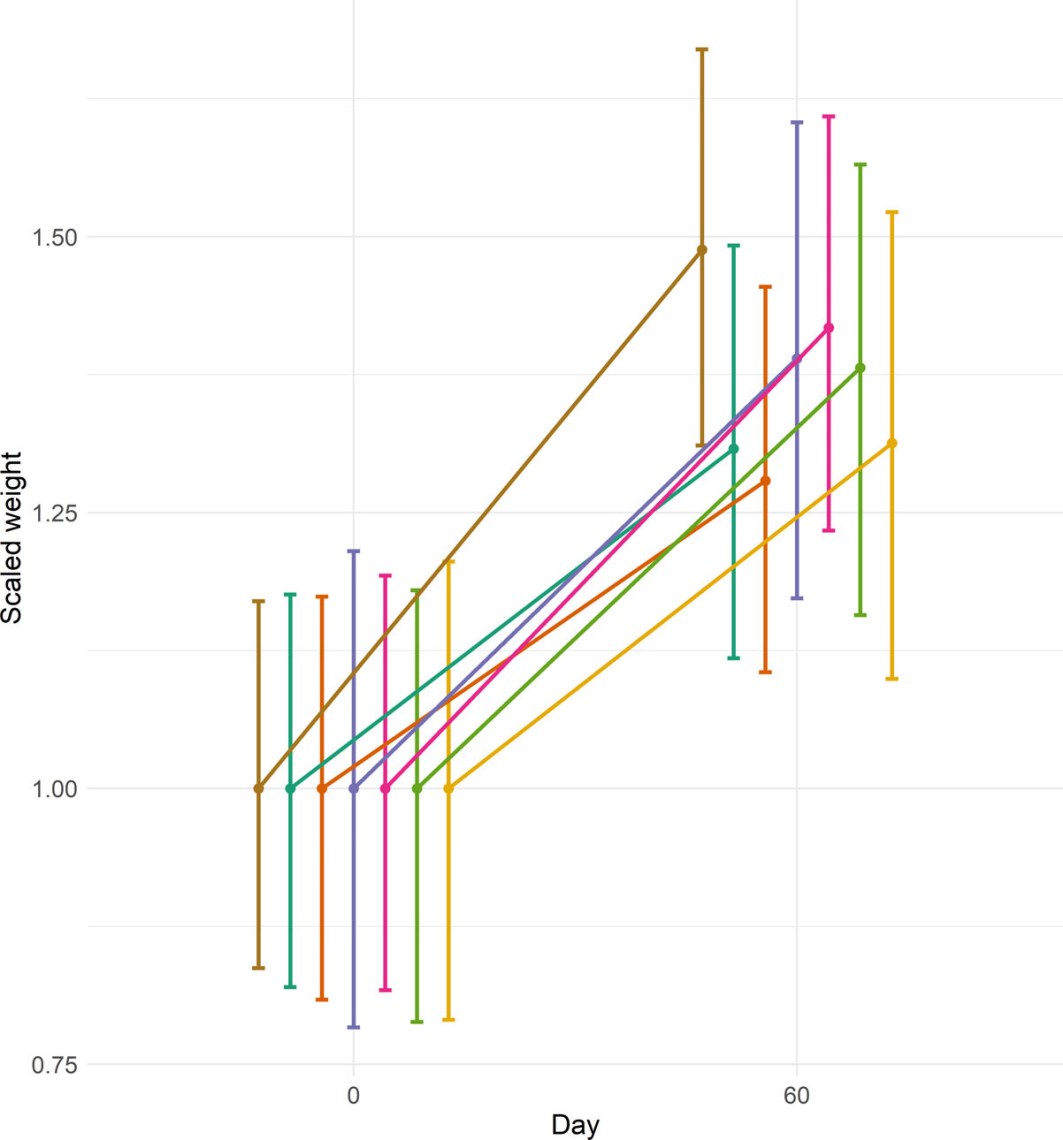
Scaled mean weights and 95% credible intervals for the interaction between *Day* and *Treatment* from the best model for predicting growth rates of *Centropristis striata* tagged with six external tag attachment methodologies and untagged control fish. Line slopes between points at day 0 and day 60 represent mean growth rates for each group. Treatment: (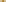) Control, (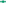) Single dart, (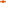) Double dart, (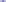) Cinch‐up, (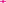) Spaghetti loop, (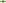) Wire, (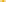) Threaded rod

Weights of *C. striata* did not differ by tagging method on the day of tagging (Supporting Information Figure S1 in Appendix S1; one‐way ANOVA *F* = 0.642, *P* = 0.70). The best model for predicting weight by LOOIC was the model containing all possible variables (Table 3). In this model, *Day* was predicted to have a positive effect on weight. The 95% credible intervals for the interaction between *Day* and treatment were less than zero for the single dart, double dart, cinch‐up, wire and threaded rod, indicating that the growth rates of fish tagged with these treatments were lower than that of untagged control fish (Supporting Information Table S1 in Appendix S1). Of the six interaction terms representing growth rate, only the term for the spaghetti loop had a 95% credible interval that overlapped with zero, indicating growth of fish tagged with this treatment was not different from growth of untagged controls. Growth rates for fish tagged with the single dart, double dart and threaded rod appeared appreciably lower than for untagged control fish (Figure 7).

**TABLE 3:** Bayesian regression models with untagged fish wet weight as the response variable. An asterisk (*) implies inclusion of both single effects and the two‐way interaction between day and treatment. LOOIC is leave‐one‐out cross validation information criterion. Tank and individual (ID) were included as random effects

Model	LOOIC	ΔLOOIC
~ Day*Trt + (1|Tank) + (1|ID)	3989.70	0.00
~ Day*Trt + (1|ID)	3991.60	1.90
~ Day + Trt + (1|ID) + (1|Tank)	4005.50	15.80
~ Day + Trt + (1|ID)	4006.50	16.80
~ Trt + (1|ID)	4501.30	511.60
~ Day + Trt + (1|Tank)	5027.00	1037.30
~ Day*Trt + (1|Tank)	5033.20	1043.50
~ Day + Trt	5060.90	1071.20
~ Trt + (1|Tank)	5065.60	1075.90
~ Day*Trt	5066.90	1077.20
~ Trt	5095.00	1105.30

## 4 DISCUSSION

Understanding the impact of tagging on wild animals is crucial to ensuring the reliability of studies of fish behaviour, movement and survival (Bridger & Booth, 2003; Jepsen *et al*., 2015). Here the authors demonstrate that several previously used methods for attaching acoustic transmitters to fish can result in adverse effects to growth and welfare, which may impact behaviour or survival thus compromising study results. Tag retention and harmful effects of tagging differed among treatments, and some methods clearly emerged as superior to others.

There was a high degree of variability in tag retention among treatments. Low retention of the single dart was likely related to the high levels of infection and inflammation observed in this group. Tag losses from the double dart were also likely a result of infection that eroded tissue and loosened dart tips. In addition to the two double‐dart‐tagged fish that fully shed their tags, one individual experienced full withdrawal of the anterior dart tip 18 days after tagging. The posterior dart remained embedded for the duration of the study. Cinch‐up tagged fish did not shed their tags, despite the incorporation of a dissolvable suture loop intended to promote tag loss. The choice of polydioxanone suture (PDS) probably led to this high level of retention. In a study comparing dissolution in water of suture loops made from poliglecaprone (Monocryl™) with an equivalent to PDS [polyglyconate (Maxon™)], Cannizzo *et al*. (2016) found poliglecaprone dissolved much faster than polyglyconate. Further, their study showed that higher water temperatures resulted in faster dissolution. Given that the authors used PDS, and water temperatures in the winter/spring study were fairly cool (range 13–20°C), future studies hoping to encourage near‐term tag loss by using suture loops should use poliglecaprone (or similar) and consider the ambient water temperatures in their study system. The authors consider the single tag loss of the spaghetti loop to be an anomaly that is unlikely to occur in the wild with proper tag application. The tag becoming untied was a result of human error: this was the first individual tagged in this group, and the knot was not tightened properly. After retying the spaghetti tag post‐tag‐shedding, this individual retained the transmitter for the remainder of the study, and none of the other eight fish in this treatment group experienced knot loosening or tag loss. Most of the tag losses from the wire treatment were a result of the brass crimp sliding off of the end of the wire; this is akin to the untying of the spaghetti tag and could possibly be corrected by modifying the crimp procedure or bending the end of the wire. Nonetheless, one individual tagged with the wire treatment experienced tag loss by the wire pulling dorsally through the musculature and eventually the dorsal fin. The full retention of the threaded rods was unsurprising due to their method of affixation using stainless steel lock nuts.

The single dart caused moderate‐to‐severe trauma to fish beginning on day 10, likely as a result of the relatively large size of the dart tip and length of the external portion of the apparatus. The swinging motion of the tag caused substantial abrasion to the lateral aspect of the fish. External trauma from the double dart was fairly low (mild‐to‐moderate) due to the two attachment points (and therefore no tag movement). The cinch‐up and spaghetti tags also caused mild‐to‐moderate trauma, though tags in these treatments were allowed to swing freely. It appears that the ability of the transmitter to slide along the flexible tag (cinch‐up or spaghetti, respectively) resulted in the difference in observed trauma from these treatments as compared to the single dart in which the tag was also free to swing but not able to slide. The spaghetti loop was the only treatment for which fish growth was decidedly not different from growth of the control group, indicating its minimal impact on fish welfare relative to the other treatments. The wire treatment caused moderate‐to‐severe trauma, largely as a result of the wire and retaining washer. The wire in this treatment was narrow and rigid enough to cut though tissue as the fish moved resulting in eventual loosening of the tag against the body of the fish. Although the credible interval for growth rates of fish tagged with the wire treatment did contain zero, the upper limit was 0.01 indicating that increased sample sizes or a study of longer duration may have resulted in an interval that did not contain zero. The trauma caused by the threaded rod was the most severe of any method examined in this study. The level of abrasion caused by the tag and associated hardware on the left (tag) side of the fish was uniformly severe from day 50 onward. Although other treatments (*e.g*., single dart) also resulted in severe trauma, the depth of the trauma caused by the threaded rod was greater and appeared to span the entire width of the fish. In addition, the washers on the right side of the fish abraded the scales and skin in most individuals. Indeed, for several fish tagged with the threaded rod, the washers became embedded sub‐dermally in muscle tissue. Across treatments, some individuals were able to heal (at least partially) at intervals throughout the study, although this never occurred for fish tagged with the threaded rod.

Though no fish died in this study, they were held in an enclosed system and were therefore not exposed to predators. Oceanic predators may preferentially feed on impaired fish (Bleckmann & Hofmann, 1999), including those that are recovering or traumatized post‐tagging (Runde *et al*., 2020). Therefore, the result of no tag‐induced mortality may not reflect what would occur in the wild, and the authors suggest that the risk of tag‐related predation probably increases with increasing tag‐related trauma. Nonetheless, they suspect this bias to be minimal, as some studies have shown no increase in predation on tagged fish (Jepsen *et al*., 2008).

Long intervals of air exposure can result in higher post‐release mortality for fish (Burns *et al*., 2002; Graves *et al*., 2016); the speed with which external transmitters can be attached is therefore an important consideration for study design. In this study, there were differences in application time (Figure 2) although almost every individual was tagged in less than 2 min regardless of treatment. The authors echo previous suggestions that researchers practice their tagging method on the study species prior to field deployments to refine mechanics and reduce tagging time as much as possible.

Values for PCV and TS were slightly lower than published values for a sympatric demersal reef fish, invasive red lionfish *Pterois volitans* L. (PCV median 34, range 27–44; TS median 4.6, range 2.5–7.5 g/dl) (Anderson *et al*., 2010), but values are comparable for *C. striata* in the same area sampled immediately following capture (CAH, unpubl. data), indicating that health was not markedly affected by the tagging systems as assessed by these nonspecific indicators. For most applications of haematology in fish studies, BC is usually negligible (0%–1%) and not reported. Larger values for BC thickness, as observed for fish tagged with the single dart (0%–4%) and threaded rod (0%–4%) in the current study, suggest an elevated white blood cell count (Kerr, 2010), consistent with infection and inflammation as also indicated by the higher external trauma scores of these groups.

Long‐term tag retention should not be an overriding goal of telemetry studies at the expense of fish welfare (Cooke *et al*., 2013; Rub *et al*., 2014). Given the low growth associated with the threaded rod, paired with the associated severe trauma, the authors cannot recommend further use of this method without substantial modification. Nonetheless, these findings pertain to black sea bass in the laboratory and further testing in the field with other species could find different results. Of the six methods used in this study, the authors recommend the spaghetti loop for use in field studies; the cinch‐up could be considered a good second choice. The high tag retention (when properly applied) combined with low external trauma and negligible effects on growth all inspire confidence that the spaghetti loop is minimally invasive and therefore ideal for use in the field. Depending on study goals, dissolvable suture loops should be incorporated into the tagging apparatus to reduce the possibility of indefinite retention of the transmitter and associated adverse physiological effects.

Some transmitters must be attached at two points in order for their sensors to function properly (Johnson *et al*., 2015; Runde *et al*., 2020), precluding the use of the spaghetti loop. The only method the authors examined that attached at two locations was the double dart, which did not perform well. Indeed, in both prior *in situ* studies using this method (Runde *et al*., 2020; Runde & Buckel, 2018) they observed several instances of tag loss. They recommend that researchers seeking a method that attaches in two places use a modification of their spaghetti loop (*e.g*., passing the spaghetti tag through the body in two places and affixing to the transmitter with heat shrink) or a method such as was used by Johnson *et al*. (2015) whereby a cinch‐up tag was passed twice through the body and attached to the transmitter *via* adhesive and plastic cable ties. Future work could compare these and other methods that attach in two places (*e.g*., Bridger & Booth, 2003; Jacobs *et al*., 2020).

A common general guideline is that electronic tags should not exceed 2% of the fish's body weight in air (Winter, 1983). Nonetheless, there is substantial evidence that tag weight:body weight ratios of over 10% can be acceptable in telemetry studies (Brown *et al*., 1999; Jepsen *et al*., 2005). In this study, ratios of tag weight (including hardware) to body weight on the day of tagging ranged from 1.7% to 6.2% (Supporting Information Figure S2 in Appendix S1). It is conceivable that the fish with a higher ratio were impaired as a result. Field studies seeking to minimize the effects of tag weight could employ smaller tags than those used here though at the potential cost of lower detection range and battery life.

The results with *C. striata* are considered mostly transferable to other demersal species, and show that some attachment methods (*e.g*., spaghetti) outperform others (*e.g*., threaded rod) in virtually every way examined. It remains unclear how these (or other) attachment methods would perform on pelagic fishes, though some results (*e.g*., among‐treatment effects on welfare) are likely to remain constant across species. Where possible, the authors recommend that researchers perform holding studies to evaluate attachment methods prior to field deployment to ensure suitability. In lieu of that, the results could be used as general guidance for field studies, including for pelagic species where holding studies may prove impractical.

## Supporting information


**APPENDIX S1** Supporting Information
