# Supplementary material for: Evaluation of six methods for external attachment of electronic tags to fish: assessment of tag retention, growth and fish welfare
Source: J Fish Biol. 2022 Jan 23;101(3):419–30. doi: 10.1111/jfb.14989 (PMC9544572; doi:10.1111/jfb.14989)
Supplement: Supplementary file 1 — APPENDIX S1 Supporting Information [file JFB-101-419-s001.docx]

Table S1. Model output from the best model (lowest LOOIC) for predicting fish weight of *C. striata* tagged with one of six transmitter attachment methods versus an untagged control. Mean coefficient estimates and 95% credible intervals are provided. ESS is effective sample size.

| Variable | Estimate | L-95% CI | I-95% CI | R-hat | ESS |
| --- | --- | --- | --- | --- | --- |
| sd(Tank) | 140.34 | 117.68 | 169.57 | 1.00 | 1543 |
| sd(ID) | 35.98 | 1.38 | 109.59 | 1.00 | 842 |
| Intercept | 376.72 | 255.28 | 492.98 | 1.00 | 2036 |
| Day | 3.38 | 2.89 | 3.86 | 1.00 | 3664 |
| TrtSingleDart | 105.71 | -27.61 | 249.62 | 1.00 | 1849 |
| TrtDoubleDart | 71.04 | -64.50 | 213.37 | 1.00 | 2011 |
| TrtCinchUp | 19.95 | -127.26 | 168.17 | 1.00 | 2032 |
| TrtSpaghetti | 85.54 | -63.93 | 229.11 | 1.00 | 1902 |
| TrtWire | 63.80 | -76.08 | 206.10 | 1.00 | 1893 |
| TrtThreadedRod | 42.13 | -106.18 | 186.67 | 1.00 | 1874 |
| Day:TrtSingleDart | -1.01 | -1.80 | -0.25 | 1.00 | 4193 |
| Day:TrtDoubleDart | -1.35 | -2.00 | -0.70 | 1.00 | 3813 |
| Day:TrtCinchUp | -0.85 | -1.46 | -0.21 | 1.00 | 3751 |
| Day:TrtSpaghetti | -0.30 | -0.94 | 0.34 | 1.00 | 3622 |
| Day:TrtWire | -0.67 | -1.35 | 0.01 | 1.00 | 3719 |
| Day:TrtThreadedRod | -1.25 | -1.87 | -0.58 | 1.00 | 3900 |


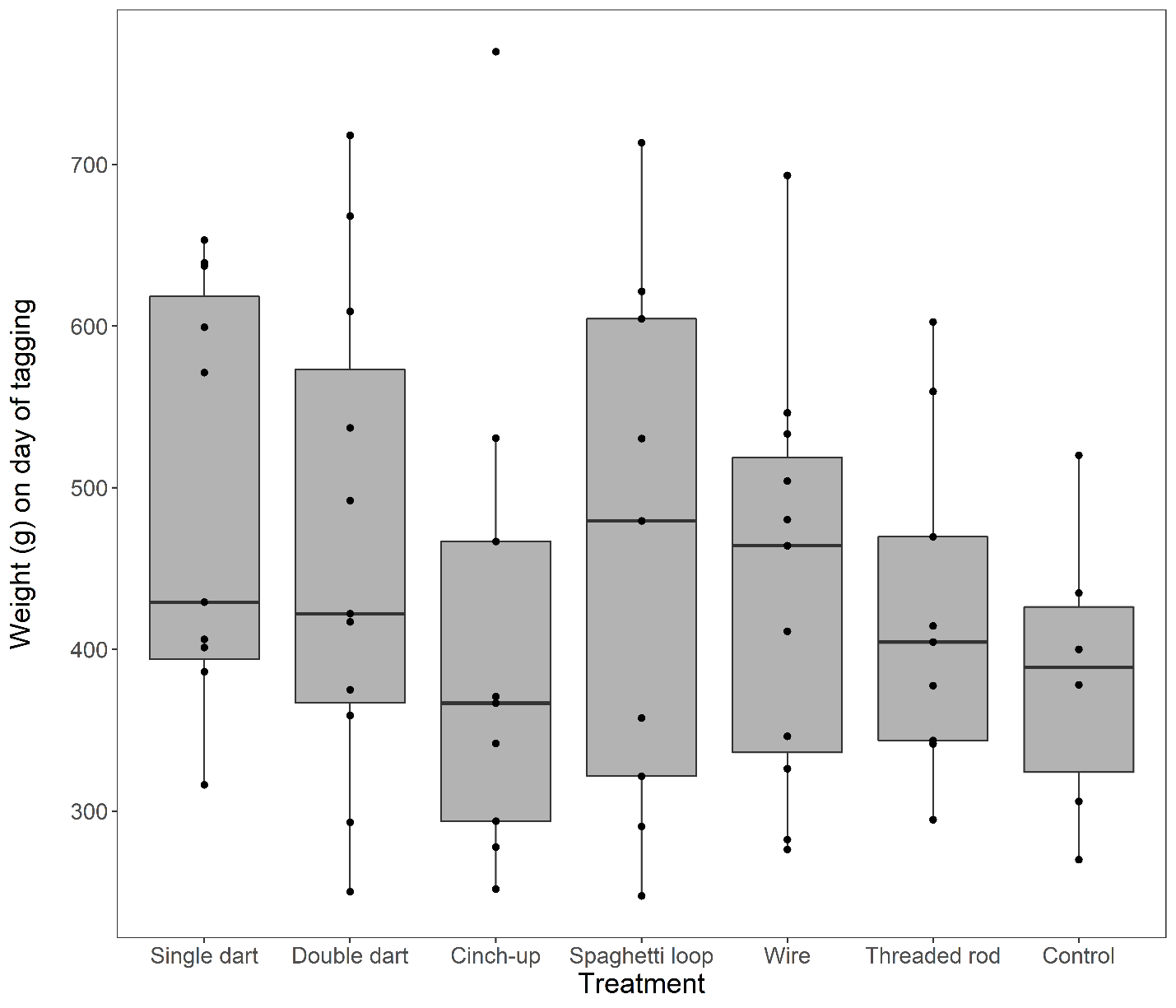


Figure S1. Weights of *C. striata* by treatment on the day of tagging. See Table 1 for a description of each method.


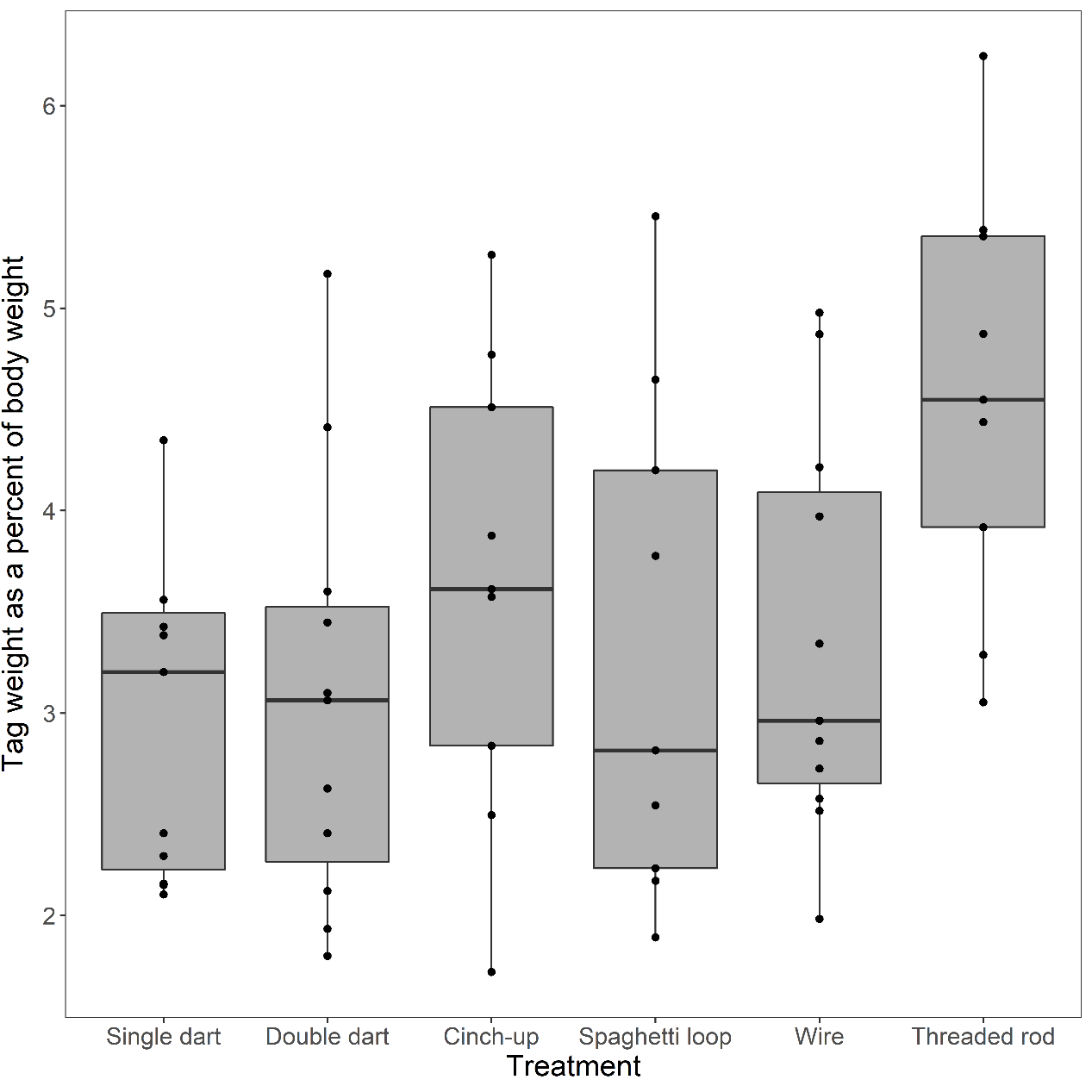


Figure S2. Tag weight in air as a percentage of body weight in air for all fish on the day of tagging. Figure S1. Weights of *C. striata* by treatment on the day of tagging. See Table 1 for a description of each method.
